# An exploratory mixed methods study on shared decision-making and antibiotic prescribing for pet cats and dogs in Singapore veterinary clinics

**DOI:** 10.1038/s41598-025-04881-w

**Published:** 2025-07-02

**Authors:** Huiling Guo, Zoe Jane-Lara Hildon, Timothy Chua, Boon Han Teo, Angela Chow

**Affiliations:** 1 Department of Epidemiology and Preventive Medicine, Office of Clinical Epidemiology, Analytics, and Knowledge, Tan Tock Seng Hospital, Singapore, Singapore; 2 Saw Swee Hock School of Public Health, National University Health System, National University of Singapore, Singapore, Singapore; 3 Singapore Veterinary Association, Singapore, Singapore; 4 Beecroft Animal Specialist & Emergency Hospital, Singapore, Singapore; 5 Lee Kong Chian School of Medicine, Nanyang Technological University, Singapore, Singapore

**Keywords:** Antimicrobial resistance, Companion animals, Veterinary clinics, Antibiotic prescribing, Shared decision-making, Health policy, Public health

## Abstract

Veterinarians primarily engage pet owners in shared decision-making (SDM) to enhance treatment outcomes and owner satisfaction, but not specifically for antibiotic stewardship. This study aims to understand how SDM occurs and how to better involve pet owners in appropriate antibiotic prescribing for their pets. This is a concurrent mixed methods study, anchoring on survey data collected from 1080 pet cat/dog owners (March─December 2023) supplemented by qualitative insights from 19 veterinarians (January─July 2022). Statistical and thematic analyses were performed. Of those who received antibiotics for their pet during the last veterinary consultation (*N* = 415), only 22.7% engaged in SDM with their veterinarians on antibiotic prescribing. Pet owners with high degree of control and empowerment to interact with veterinary professionals were more likely to do so. Veterinarians were capable of engaging pet owners in SDM; exploring owners’ needs and feeding concerns was key. Pet owners trusted veterinarians’ final antibiotic decisions and desired appropriate antibiotic prescriptions from them, alongside training and advice on how best to medicate their pets with antibiotics. Proactive exploration of pet owners’ needs and concerns, and providing pet owners with necessary training and advice, can enhance antibiotic stewardship for pet cats and dogs.

**Supplementary Information:**

The online version contains supplementary material available at 10.1038/s41598-025-04881-w.

## Introduction

The silent pandemic of antimicrobial resistance (AMR) threatens the human population with an estimated 10 million annual global deaths by 2050^1^. Concerted efforts by the human, animal and environmental health sectors are critical^2^, given the potential for AMR to spread between these sectors^3^.

Compared to existing antibiotic regulations for food-producing animal husbandry^4^, regulatory control on how antibiotics are prescribed for companion animals, or pets, is often less strict. This is in spite of pet owners being at a constant risk of exposure to resistant pathogens that are common between humans, cats and dogs^5–7^. Such risks are contributed by prolonged intimate behaviours with these animals in confined living spaces^8^, and compounded by the common use of third and fourth generation cephalosporins for feline and canine treatments^9–11^. There is therefore a need to promote prudent antibiotic use in the veterinary setting. Evidence-based interventions addressing the key determinants of inappropriate antibiotic use amongst pet cats and dogs are essential^12,13^.

Shared decision-making (SDM) is a successful strategy that can reduce inappropriate antibiotic prescribing for patients in the human primary care setting^14^, by addressing expectations and demands for unnecessary antibiotics^15^. Through the process of SDM^16^, together with identifying and addressing personal values deeply cherished by the patient and doctor^17^, undesirable antibiotic-related behaviours can be avoided. This was highlighted in the veterinary setting as well^18^. However, the concept of SDM for antibiotic stewardship in the veterinary setting has not been explored purposefully thus far, although SDM for general animal care^19,20^ is reportedly common and has achieved good owners’ satisfaction levels^21,22^.

In this study, we aimed to understand SDM in veterinary setting better. Our objectives were to (1) identify pet owner’s attitudes towards antibiotic administration for their pets; (2) assess typical encounters of pet owners with SDM and contrasting these with veterinarians’ views of when, how and why these decisions tend to occur; and (3), finally, account for the role of empowerment in SDM, against the backdrop of these earlier analyses.

## Methods

### Study design and participants

This is a concurrent mixed methods study, comprising of a dominant quantitative survey of pet owners and qualitative interviews with veterinarians to explain the survey findings. Cat and dog owners, aged 21 years and above and attending at veterinary clinics stratified by practice size (solo/small group and large chain) and at different locations in Singapore (North/South/East/West/Central), were invited to complete a self-administered survey between March and December 2023. These pet owners were approached while waiting for their pet’s veterinary consultation at each clinic’s designated holding area. Those who agreed to take part were asked to complete an online questionnaire via the study team’s tablet or via their own mobile devices.

Veterinarians were purposively sampled from licensed veterinary clinics of varying practice sizes in Singapore and invited to take part in an in-depth interview between January and July 2022. A good representation of both genders recruited for the interviews was ensured and sampling was anchored by principles of data saturation^23^. This study was approved by the Domain Specific Review Board, National Healthcare Group Singapore.

### Survey instrument and variable selection

The survey instrument (Supplementary Material 1) was designed with close-ended questions assessing the level of SDM and empowerment in pet owners on the antibiotic treatments prescribed for their pets, using Kriston et al.’s SDM-Q-9 scale^24^, presented in a 5-point Likert scale (1-Strongly disagree to 5-Strongly agree), and Gagnon et al.’s HCEQ-10 scale^25^, presented in a 4-point Likert scale (1-Not at all/Not important at all to 4-Extremely/Extremely important), respectively. Both scales were reworded to fit the veterinary context. Additionally, to explore the pet owners’ attitudes towards antibiotic administration for their pets, close-ended questions reported in existing literature^26,27^ were included in a 5-point Likert scale (1-Strongly disagree to 5-Strongly agree) as well. Further socio-demographic information were collected and analysed alongside, and the survey was available in English language only.

Under the modified SDM-Q-9 scale, pet owners who agreed/strongly agreed with each of the 9 statements were categorised as having that component of SDM occurring with their veterinarians. Overall, pet owners who agreed/strongly agreed with all 9 statements under the modified scale were categorised as having *Engaged in SDM* with their veterinarians. For the modified HCEQ-10 scale, composite scores were tabulated for each dimension: involvement in decisions, involvement in interactions, and degree of control, according to the original literature^25^. A ≥ 75% distribution threshold was used to define pet owners as having a *High Level of Empowerment in Involvement in Decisions* (score of ≥ 21 out of 24), a *High Level of Empowerment in Involvement in Interactions* (score of ≥ 28 out of 32), and a *High Level of Empowerment in Degree of Control* (score of ≥ 18 out of 24). These nomenclatures are further shortened to *“high empowerment in antibiotic decisions”*, *“high empowerment to interact”* and *“high degree of control”* in later text. Lastly, responses on pet owners’ attitudes towards antibiotic administration for their pets were dichotomised into two categories: strongly agree and agree were combined and referred to as “assent” and the remaining categories (neither agree nor disagree, disagree and strongly disagree) were combined and referred to as “non-assent”.

### Quantitative data analysis

Categorical and dichotomised variables were presented as proportions, and Chi-squared test was applied to compare any differences. Multivariable logistic regression was performed to determine the independent factors associated with pet owners’ engagement in SDM on antibiotic prescribing with their veterinarian during the last veterinary consult. Covariates were selected through assessing the Akaike information criteria, Bayesian information criteria and likelihood ratios, and included in the final regression model to adjust for potential confounding. Statistical significance was benchmarked as *P*-value < 0.05 and statistical analyses were conducted in Stata version 14.0 (StataCorp LLC, College Station, Texas US).

### In-depth interviews

The design of the interview guide was anchored on a VALUE model^28^ to understand the context and mechanisms influencing antibiotic prescribing for cats and dogs in the veterinary clinics. Only the responses related to questions pertaining to *Liaison with Pet Owners* were included to supplement the quantitative analysis presented in this paper. These interview questions were: “In this clinic you are practising in, is there an emphasis on shared decision-making with pet owners on antibiotic prescribing?”, “Would you think that it is necessary for shared decision-making to take place with pet owners on antibiotic prescribing?”, and “Do you feel that pet owners would prefer shared decisions for antibiotics?”.

All interviews were conducted virtually by HG (PhD, Female, Epidemiologist) and another Research Assistant, who were both trained in qualitative methods. Every interview lasted around 60 min, was audio-recorded and transcribed verbatim.

### Qualitative data analysis

Deductive thematic analysis was applied^29^ to explore veterinarians’ engagement or non-engagement in SDM with pet owners on antibiotic prescribing for their pets, using the COM-B model under the Behaviour Change Wheel^30^ as coding framework. Atlas.ti 9 was used to manage the data, house the coding and record emergent themes. All transcripts were independently coded by HG, and the codes were agreed by lead authors. The broader themes are reported in **bold**.

## Results

### Basic characteristics of participants

The basic characteristics of the 1080 pet owners who responded to the survey and the 19 veterinarians who took part in the interviews were described in Tables 1 and 2 respectively.

**Table 1:** Basic characteristics of pet owners recruited from veterinary clinics in Singapore.

Characteristics	Total (*N* = 1080)	Own cat(s) (*N* = 408)	Do not own any cat (*N* = 672)
Age group, N(%)			
21–34 years	399 (36.9)	167 (40.9)	232 (34.5)
35–49 years	382 (35.4)	145 (35.5)	237 (35.3)
≥ 50 years	299 (27.7)	96 (23.5)	203 (30.2)
Gender, N(%)			
Male	390 (36.1)	133 (32.6)	257 (38.2)
Female	690 (63.9)	275 (67.4)	415 (61.8)
Ethnic group, N(%)			
Chinese	798 (73.9)	237 (58.1)	561 (83.5)
Malay	91 (8.4)	90 (22.1)	1 (0.2)
Indian	70 (6.5)	22 (5.4)	48 (7.1)
Others	121 (11.2)	59 (14.5)	62 (9.2)
Educational level, N(%)			
Lower educated (GCE A-Level and below)	141 (13.1)	51 (12.5)	90 (13.4)
Higher educated (Diploma and above)	939 (86.9)	357 (87.5)	582 (86.6)
Years of experience as a cat/dog owner, N(%)			
Less than 10 years	535 (49.5)	225 (55.2)	310 (46.1)
10 years and above	545 (50.5)	183 (44.9)	362 (53.9)
Received antibiotics for pet during last veterinary consult, N(%)			
Yes	415 (38.4)	162 (39.7)	253 (37.7)

**Table 2:** Basic characteristics of veterinarians who participated in the in-depth interviews.

Characteristics	Total (*N* = 19)
Age, years	
Median (min, max)	36 (24,58)
Gender, N(%)	
Male	6 (32)
Female	13 (68)
Highest educational level, N(%)	
Basic veterinary degree	14 (74)
Post-graduate qualification	5 (26)
Employment status, N(%)	
Full-time permanent	19 (100)
Years in veterinary practice, N(%)	
≤ 10 years	9 (47)
> 10 years	10 (53)
Years in current practice, N(%)	
≤ 10 years	14 (74)
> 10 years	5 (26)

### Pet owners’ attitudes towards antibiotic administration for their pets

**Fig. 1:**
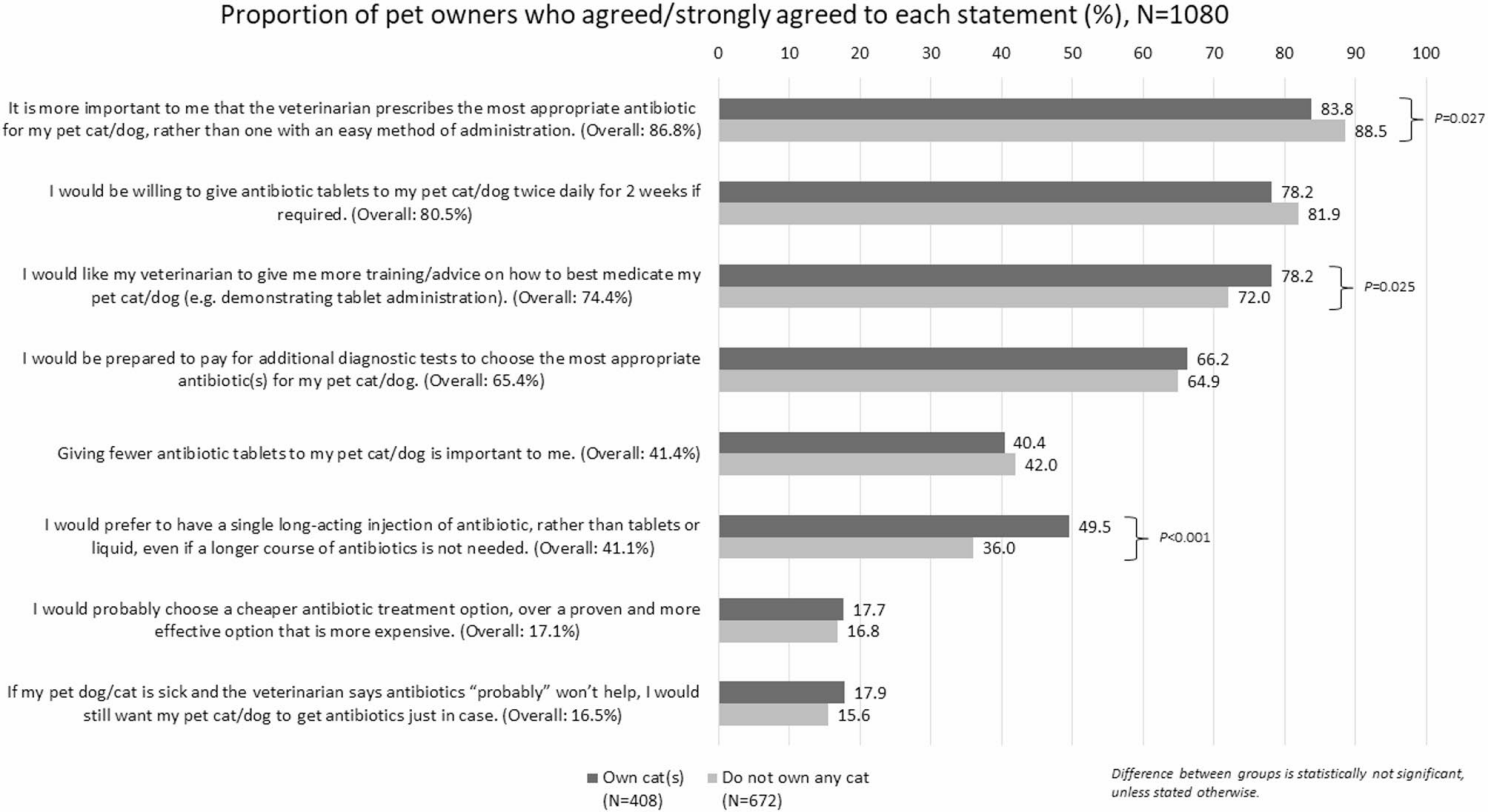
Pet owners’ attitudes towards antibiotic administration for their pets.

Most pet owners felt that it was more important that their veterinarian prescribed the most appropriate antibiotic for their pet than an antibiotic that was easier to administer (86.8%) (Fig. 1). Nearly three-quarters of them would like their veterinarians to provide more training and advice on how to best medicate their pets, which included demonstrating on how to administer the tablet (74.4%). Cat owners were more likely to desire training and advice to administer antibiotics to their pets (78.2% vs. 72.0%, *P* = 0.025), and were also more likely to prefer a single long-acting injection of antibiotic, rather than tablets or liquid, even if a longer course of antibiotics was not needed (49.5% vs. 36.0%, *P* < 0.001).

### Shared decision-making between pet owners and veterinarians on antibiotic prescribing for their pets

**Fig. 2:**
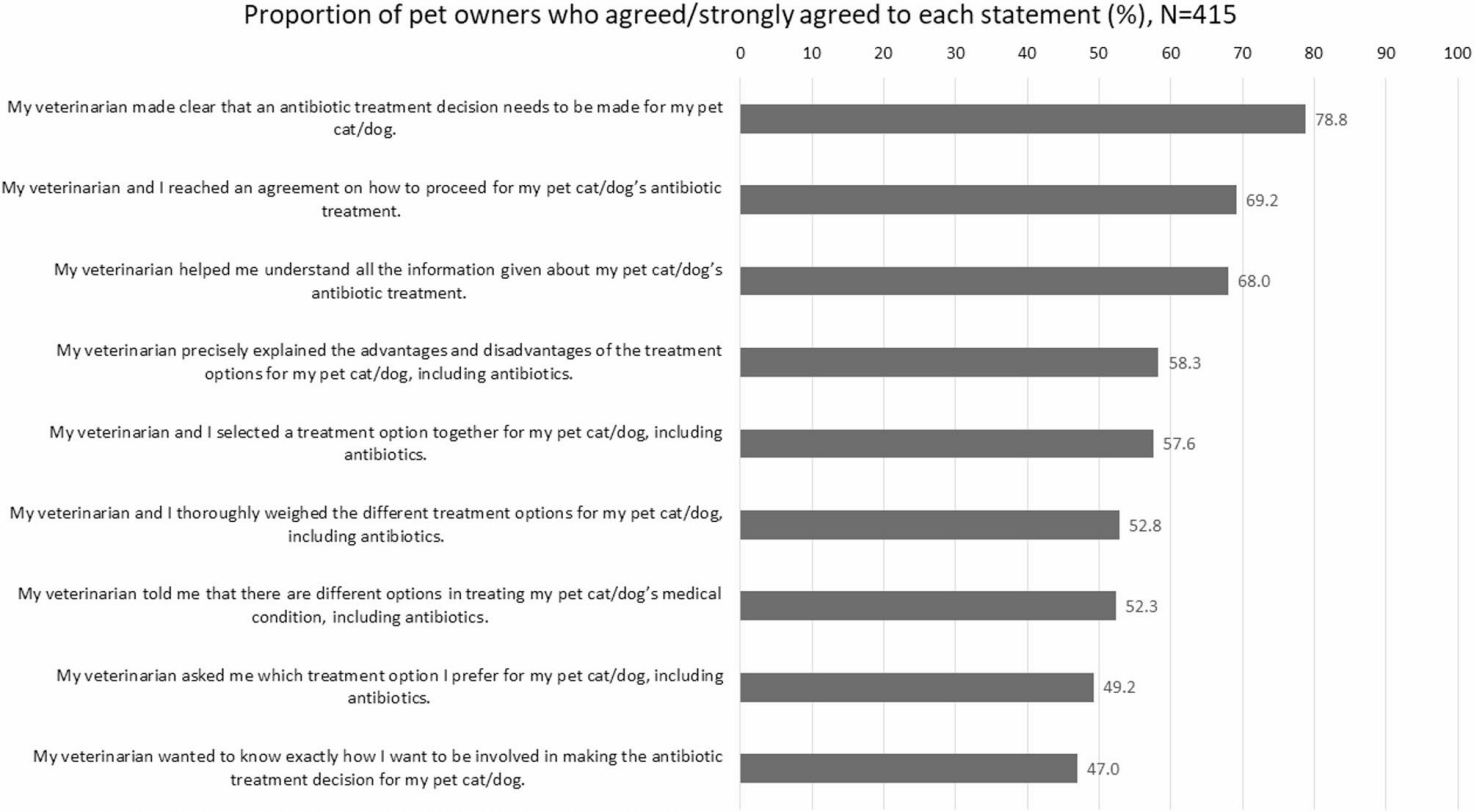
Shared decision-making between pet owners and veterinarians on antibiotic prescribing for pet cats and dogs during the last veterinary consult.

Out of the 415 pet owners who received antibiotics for their pet cat or dog during the last veterinary consult, only 94 (22.7%) reported engaging in SDM on antibiotic prescribing with their veterinarian. Veterinarians shared that **SDM was rarely a practice protocol in veterinary clinics but usually driven by individual practitioner’s own initiative**. Veterinarians’ capability to engage in SDM was supported by both quantitative and qualitative findings. Pet owners reported being told by their veterinarians that there were different treatment options for their pet cat/dog’s medical condition, including antibiotics (52.3%), and that their veterinarians precisely explained the advantages and disadvantages of the treatment options (58.3%), selected a treatment option (57.6%) and thoroughly weighed the different treatment options (52.8%) together with them (Fig. 2). However, less than half of the pet owners were asked by their veterinarians about their preferred treatment option for their pet cat/dog (49.2%) and how they wanted to be involved in making the antibiotic treatment decision for their pet cat/dog (47.0%).

This phenomenon was explained by a couple of veterinarians, highlighting that **SDM was typical for *“a discussion on how the pet owners would like to administer the antibiotic*,* but there would never be discussions on what they would like to have”*** (VP03). This was due to the potential dangers of *“getting lost in trying to please the pet owner and lose the science behind antibiotic prescribing”* (VP03), as pet owners were perceived to have insufficient knowledge to take part in such conversations. Conversely, interviewees felt that pet owners would also trust the veterinarians to make the final decision, as the pet owners *“do not know what is best for the animal”*, and they would like the veterinarian to *“give their firm opinion”* instead (VP06).

Nonetheless, **the key motivation that could potentially underpin SDM was the belief that it is** ***“the right of the owner to choose the option that best fits in their circumstances”*** (VP19), which includes pet owners’ ability to administer the antibiotic, willingness to pay for diagnostic tests that may lead to better antibiotic choices, and affordability of the antibiotic prescribed. Most importantly, as expressed by the interviewees, while SDM *“creates a collaboration between the veterinarian and the owner”* (VP06), *“at the end of the day*,* even though the pet is a living thing*,* it is still a property owned by the owner*,* who makes the ultimate decision of what needs to be done”* (VP01). Furthermore, some veterinarians also emphasised that they would avoid making and forcing unilateral decisions onto pet owners, for fear that *“if things do not turn out well*,* the pet owners would come back and write them a complaint letter”* (VP19).

Very often, SDM occurred to address antibiotic administration concerns by pet owners, for example discussing *“the choice of dosage*,* and form of administration such as injectable versus oral*,* or topical based on what the pet owners feel that it would be most successful in getting the most compliance from their pets”* (VP18). However, the opportunities for these discussions could be impeded. **Veterinarians would** ***“trust that pet owners will give them that information”*** **on any antibiotic administration concerns to initiate the conversation (VP03); otherwise, SDM might not occur**. In addition, the surprisingly common situation in Singapore where pet owners do not attend the veterinary consultation personally but are represented by another individual from the household was mentioned as well, limiting opportunities for effective communications between veterinarians and pet owners at the point of consultation. In some circumstances, veterinarians were also required to *“reschedule a visit in two weeks’ time*,* to suit the time of the pet owner whom they want to see”* (VP10) for successful SDM to happen.

### Empowerment of pet owners in relation to antibiotic treatments for their pets

After adjusting for potential confounders, cat owners (aOR 1.96, 95% CI 1.08–3.57, *P* = 0.027) were almost twice as likely as non-cat owners, to engage in SDM with their veterinarians on antibiotic prescribing during the last veterinary consultation (Table 3). Notably, those with a high empowerment to interact with veterinary professionals (aOR 3.76, 95% CI 1.25–11.28, *P* = 0.018), and those with a high degree of control on the antibiotic treatment and services received by their pets (aOR 4.00, 95% CI 2.30–6.95, *P* < 0.001) were four times as likely as those who had a low level of empowerment, to engage in SDM with their veterinarians on antibiotic prescribing during the last veterinary consultation. There was no evidence of an association between having a high empowerment in antibiotic decisions for their pets and engagement in SDM (aOR 1.01, 95% CI 0.34-3.00, *P* = 0.982).

**Table 3:** Univariate and multivariable analyses to assess the factors influencing shared decision-making between pet owners and veterinarians on antibiotic prescribing during the last veterinary consult.

Variables	Total (*N* = 415)	Engaged in SDM (*N* = 94)	Did not engage in SDM (*N* = 321)	*P*-value	Univariate analysis (*N* = 415)		Multivariable analysis* (*N* = 415)	
					Odds ratio (95% CI)	*P*-value	Adjusted odds ratio (95% CI)	*P*-value
Own cat(s), N(%)								
Yes	162 (39.0)	45 (47.9)	117 (36.5)	**0.046**	1.60 (1.01–2.55)	**0.047**	1.96 (1.08–3.57)	**0.027**
Involvement in antibiotic decisions, N(%)								
High-level of empowerment	119 (28.7)	50 (53.2)	69 (21.5)	**< 0.001**	4.15 (2.56–6.74)	**< 0.001**	1.01 (0.34-3.00)	0.982
Involvement in interactions with veterinary professionals, N(%)								
High-level of empowerment	107 (25.8)	51 (54.3)	56 (17.5)	**< 0.001**	5.61 (3.41–9.23)	**< 0.001**	3.76 (1.25–11.28)	**0.018**
Degree of control in regard to antibiotic treatment and services received for their pets, N(%)								
High-level of empowerment	118 (28.4)	55 (58.5)	63 (19.6)	**< 0.001**	5.78 (3.52–9.47)	**< 0.001**	4.00 (2.30–6.95)	**< 0.001**

*Adjusted for age, gender, ethnic group, educational level, and years of experience as a cat/dog owner. Bold values indicate statistical significance of *P* < 0.05.

## Discussion

This study has provided valuable insights on the occurrence of SDM in the veterinary setting and how SDM can be leveraged to promote appropriate antibiotic prescribing for pet cats and dogs. Overall, SDM on antibiotic prescribing was not a common practice in the veterinary clinics in Singapore but veterinarians were reportedly capable of engaging pet owners in making shared decisions, with more than half of pet owners responding that their veterinarian shared different treatment options, explained and weighed each treatment option, and selected a treatment option, together with them. These existing skills of veterinarians to engage in SDM with pet owners could be attributed by the emphasis of SDM and effective communications for the purpose of improving clinical outcomes in general animal care^19,20^.

Addressing antibiotic-related concerns by pet owners, i.e. finding an optimal antibiotic treatment option that fits the pet owners’ lifestyle, finances and ability to medicate, is critical in most decision-making processes for pets when antibiotic treatments are needed^31^, and it was also evident in this study. Empowering pet owners to be part of the dialogue to better understand the need, the type and the amount of antibiotics to be prescribed for their pets promotes SDM (aOR 4.00), highlighting the value of involving pet owners in these discussion topics although these are professional decisions to be made by veterinarians. Even though the key motivation for veterinarians to engage in SDM with pet owners on antibiotic prescribing was not for antibiotic stewardship but to honour the rights of ownership and to avoid unnecessary complaints, these are existing SDM opportunities to explain the appropriateness or inappropriateness of antibiotic treatments to pet owners. With the majority of the pet owners (86.8%) perceiving that it was important that their pets be prescribed the most appropriate antibiotics and many of them opining that they trusted their veterinarians to make the final antibiotic decisions on their behalf due to perceived poor personal knowledge on antibiotic use^32^, counselling pet owners against undesirable antibiotic behaviours seems to have minimal impact on their satisfaction levels.

Pet owners often see themselves as advocates for their pets and they desire to actively participate in the decision-making processes for their pets, by being allowed to share their perspectives of their pets’ conditions with the veterinarians, be validated by the veterinarians and receive answers to the questions that they may have^33^. High empowerment to interact with veterinary professionals on their pet’s antibiotic treatment predicts SDM (aOR 3.76), but only 25.8% of pet owners reported such level of empowerment. This could potentially be due to a paternalistic relationship between veterinarians and pet owners, which the latter may suppress their thoughts pertaining to their pet’s antibiotic treatment from their veterinarians. Since the onus was on the pet owners to initiate such conversations, there is a need for interventions to encourage proactive interactions with veterinarians. Interventions should include providing opportunities for pet owners to share any concerns that they may have with regard to the antibiotic treatment options offered to their pets. Visual cues such as posters on the walls of consultation rooms or stickers on consultation tables could nudge both veterinarians and pet owners to engage in these active conversations. Under circumstances whereby the pet owner cannot be present personally at the consultation and is represented by a third party, teleconferencing options could be made available for veterinarians to speak with the pet owners directly and engage in SDM with them virtually.

Out of convenience and compliance by pet owners to the prescribed antibiotic feeding regimen while avoiding aggressive behaviours that may inflict accidental injuries to both the animals and their owners^34,35^, cats are more likely to be prescribed long-acting injectable antibiotics such as cefovecin, a third-generation cephalosporin. This was also observed in our study that nearly half of the cat owners surveyed would prefer a single long-acting injection of antibiotic, rather than tablets or liquids (49.5%). However, veterinarians may have underestimated cat owners’ ability to medicate their pets, hence resulting in cefovecin being unnecessarily prescribed^36^. Around the world, resistance against cefovecin was already detected in 10–20% of *Escherichia coli* strains isolated from cats and dogs^37,38^, calling for prudent use of this antibiotic.

Interestingly, cat owners were more likely to engage in SDM with veterinarians for antibiotic prescribing (aOR 1.96) and 78.2% of cat owners were in favour of receiving training or advice from their veterinarians on how to best medicate their pet cats. Hence, this suggests that addressing cat owners’ medication administration concerns could support antibiotic stewardship. Allocating time by veterinarians to train owners on how to best administer antibiotic tablets or liquids to their pet cats, through demonstrations and hands-on practice, could empower the cat owners in successfully medicating their pets. This could potentially reduce the use of cefovecin for the sole purpose of providing ease and convenience for cat owners. In view of time constraints experienced by veterinarians due to high clientele load, such trainings could also be alternatively provided by veterinary nurses or veterinary technicians working in the same clinic as well.

Based on our knowledge, this is the first ever study conducted in a developed Asian country to explore SDM for the purpose of antibiotic prescribing for pet cats and dogs in veterinary clinics. The use of mixed methods has brought further clarity to explain when and how pet owners were engaged in SDM on antibiotic prescribing, through the lens of both the veterinarians and pet owners. Purposive sampling with maximum variation was undertaken for both the quantitative survey and the qualitative interviews to ensure representativeness amongst the pet owners and veterinarians, respectively. Nonetheless, even though the survey was anonymous and the interviews were conducted in private and confidential settings, social desirability bias, though minimal, might exist. Lastly, there could be unknown confounders that were not adjusted for in the final logistic regression model.

## Conclusions

Veterinarians engaged pet owners in SDM on antibiotic prescribing, and they were trusted by pet owners for their final antibiotic decisions. Proactive exploration of pet owners’ needs and concerns during SDM, and providing pet owners with the necessary training and advice, can enhance antibiotic stewardship for pet cats and dogs.

## Electronic supplementary material

Below is the link to the electronic supplementary material.

Supplementary Material 1

## Data Availability

The data that support the findings of this study are available from Department of Epidemiology and Preventive Medicine, Office of Clinical Epidemiology, Analytics, and Knowledge, Tan Tock Seng Hospital (TTSH), Singapore, but restrictions apply to the availability of these data, so are not publicly available. The data are however available upon reasonable request to authors. For enquiries please can contact Dr Guo Huiling, email: Huiling_GUO@ttsh.com.sg.

## Abbreviations

AMR: Antimicrobial resistance
SDM: Shared decision-making
