# Supplementary material for: An exploratory mixed methods study on shared decision-making and antibiotic prescribing for pet cats and dogs in Singapore veterinary clinics
Source: Sci Rep. 2025 Jul 2;15:23135. doi: 10.1038/s41598-025-04881-w (PMC12218086; doi:10.1038/s41598-025-04881-w)
Supplement: Supplementary file 1 — Supplementary Material 1 [file 41598_2025_4881_MOESM1_ESM.pdf]

Q1. How long have you been a cat/dog owner? ___________________years___________________months

Q2. How many pet cats/dogs do you <u>currently</u> own?

Cats: ___________________

Dogs: ___________________

For the following questions, please consider your pet's ***most recent consultation with the veterinary practitioner (vet)***.

Q3. Did your pet cat/dog receive antibiotics from the veterinary practitioner (vet) during that consultation?

Please tick ONE response

Yes	☐ 1
No (Skip to Q7)	☐ 2
Cannot remember (Skip to Q7)	☐ 98

Q4. On the scale shown below, how much do you agree with the following statements related to the decision-making in your pet cat/dog's last consultation with the veterinary practitioner (vet)?

	**Strongly Disagree**	**Disagree**	**Neither Agree Nor Disagree**	**Agree**	**Strongly Agree**
a) My veterinary practitioner (vet) made clear that an antibiotic treatment decision needs to be made for my pet cat/dog	☐ 1	☐ 2	☐ 3	☐ 4	☐ 5
b) My veterinary practitioner (vet) wanted to know exactly how I want to be involved in making the antibiotic treatment decision for my pet cat/dog	☐ 1	☐ 2	☐ 3	☐ 4	☐ 5
c) My veterinary practitioner (vet) told me that there are different options in treating my pet cat/dog's medical condition, including antibiotics	☐ 1	☐ 2	☐ 3	☐ 4	☐ 5
d) My veterinary practitioner (vet) precisely explained the advantages and disadvantages of the treatment options for my pet cat/dog, including antibiotics	☐ 1	☐ 2	☐ 3	☐ 4	☐ 5
e) My veterinary practitioner (vet) helped me understand all the information given about my pet cat/dog's antibiotic treatment	☐ 1	☐ 2	☐ 3	☐ 4	☐ 5
f) My veterinary practitioner (vet) asked me which treatment option I prefer for	☐ 1	☐ 2	☐ 3	☐ 4	☐ 5

my pet cat/dog, including antibiotics					
g) My veterinary practitioner (vet) and I thoroughly weighed the different treatment options for my pet cat/dog, including antibiotics	☐ 1	☐ 2	☐ 3	☐ 4	☐ 5
h) My veterinary practitioner (vet) and I selected a treatment option together for my pet cat/dog, including antibiotics	☐ 1	☐ 2	☐ 3	☐ 4	☐ 5
i) My veterinary practitioner (vet) and I reached an agreement on how to proceed for my pet cat/dog's antibiotic treatment	☐ 1	☐ 2	☐ 3	☐ 4	☐ 5

Q5. On the scale below, to what extent was each of the following statements actualized during your pet cat/dog's most recent consultation with the veterinary practitioner (vet)?

	**Not At All**	**Somewhat**	**Very Much**	**Extremely**
a) I was able to ask for explanations regarding the use of antibiotics for my pet cat/dog's condition	☐ 1	☐ 2	☐ 3	☐ 4
b) I was able to ask questions regarding the use of antibiotics for my pet cat/dog's condition	☐ 1	☐ 2	☐ 3	☐ 4
c) I was able to ask for advice regarding the use of antibiotics for my pet cat/dog's condition	☐ 1	☐ 2	☐ 3	☐ 4
d) I was able to talk to a veterinary practitioner (vet), veterinary technician or veterinary nurse to answer my questions pertaining to the antibiotic treatment for my pet cat/dog	☐ 1	☐ 2	☐ 3	☐ 4
e) My choices for my pet cat/dog's antibiotic treatment were respected	☐ 1	☐ 2	☐ 3	☐ 4
f) I was able to obtain all the information I want with regard to the antibiotic treatment for my pet cat/dog	☐ 1	☐ 2	☐ 3	☐ 4
g) I had gotten all the help my pet cat/dog needs for its antibiotic treatment	☐ 1	☐ 2	☐ 3	☐ 4
h) My pet cat/dog's carers (e.g. family members, friend, partner) and I decided the need for the antibiotic treatment and services for my pet cat/dog	☐ 1	☐ 2	☐ 3	☐ 4
i) My pet cat/dog's carers (e.g. family members, friend, partner) and I decided the type of antibiotic treatment and services received by my pet cat/dog	☐ 1	☐ 2	☐ 3	☐ 4
j) My pet cat/dog's carers (e.g. family	☐ 1	☐ 2	☐ 3	☐ 4

members, friend, partner) and I decided the amount of antibiotic treatment and services for my pet cat/dog

Q6. On the scale below, to what extent are the following statements <u>important</u> to you, during your pet cat/dog's most recent consultation with the veterinary practitioner (vet)?

	**Not Important at All**	**Slightly Important**	**Very Important**	**Extremely Important**
a) I am able to ask for <u>explanations</u> regarding the use of antibiotics for my pet cat/dog's condition	☐ 1	☐ 2	☐ 3	☐ 4
b) I am able to ask <u>questions</u> regarding the use of antibiotics for my pet cat/dog's condition	☐ 1	☐ 2	☐ 3	☐ 4
c) I am able to ask for <u>advice</u> regarding the use of antibiotics for my pet cat/dog's condition	☐ 1	☐ 2	☐ 3	☐ 4
d) I am able to talk to a veterinary practitioner (vet), veterinary technician or veterinary nurse to answer my questions pertaining the antibiotic treatment for my pet cat/dog	☐ 1	☐ 2	☐ 3	☐ 4
e) My choices for my pet cat/dog's antibiotic treatment are respected	☐ 1	☐ 2	☐ 3	☐ 4
f) I am able to obtain all the information I want with regard to the antibiotic treatment for my pet cat/dog	☐ 1	☐ 2	☐ 3	☐ 4
g) I get the help my pet dog/cat needs for its antibiotic treatment	☐ 1	☐ 2	☐ 3	☐ 4
h) My pet cat/dog's carers (e.g. family members, friend, partner) and I decide the need for the antibiotic treatment and services for my pet cat/dog	☐ 1	☐ 2	☐ 3	☐ 4
i) My pet cat/dog's carers (e.g. family members, friend, partner) and I decide the type of antibiotic treatment and services received by my pet cat/dog	☐ 1	☐ 2	☐ 3	☐ 4
j) My pet cat/dog's carers (e.g. family members, friend, partner) and I decide the amount of antibiotic treatment and services for my pet cat/dog	☐ 1	☐ 2	☐ 3	☐ 4

Q7. If your pet dog/cat were to require antibiotics for its treatment, please indicate on the scale below, how much you agree with the following statements?

	**Strongly Disagree**	**Disagree**	**Neither Agree Nor Disagree**	**Agree**	**Strongly Agree**
a) It is more important to me that the veterinary practitioner (vet) prescribes the most appropriate antibiotic for my pet cat/dog, rather than one with an easy	☐ 1	☐ 2	☐ 3	☐ 4	☐ 5

	method of administration					
b)	I would be willing to give antibiotic tablets to my pet cat/dog twice daily for 2 weeks if required	☐ 1	☐ 2	☐ 3	☐ 4	☐ 5
c)	Giving fewer antibiotic tablets to my pet cat/dog is important to me	☐ 1	☐ 2	☐ 3	☐ 4	☐ 5
d)	I would prefer to have a single long-acting injection of antibiotic, rather than tablets or liquid, even if a longer course of antibiotics is not needed	☐ 1	☐ 2	☐ 3	☐ 4	☐ 5
e)	I would be prepared to pay for additional diagnostic tests to choose the most appropriate antibiotic(s) for my pet cat/dog	☐ 1	☐ 2	☐ 3	☐ 4	☐ 5
f)	I would like my veterinary practitioner (vet) to give me more training/advice on how to best medicate my pet cat/dog (e.g. demonstrating tablet administration)	☐ 1	☐ 2	☐ 3	☐ 4	☐ 5
g)	I would probably choose a cheaper antibiotic treatment option, over a proven and more effective option that is more expensive	☐ 1	☐ 2	☐ 3	☐ 4	☐ 5
h)	If my pet dog/cat is sick and the veterinary practitioner (vet) says antibiotics "probably" won't help, I would still want my pet cat/dog to get antibiotics just in case	☐ 1	☐ 2	☐ 3	☐ 4	☐ 5

We need to find out a little about your background to help us better understand the information you shared with us. All detail in this questionnaire will be kept strictly confidential.
Please ***TICK*** *the response category that applies to you.*

Q8. What is your birth year? ___________________

Q9. What is your ethnicity?

	**Please tick ONE response**
Chinese	☐ 1
Malay	☐ 2
Indian	☐ 3
Others	☐ 4

Q10. What is your gender?

	**Please tick ONE response**
Female	☐ 1
Male	☐ 2

Q11. What is your highest educational qualification?

Please tick ONE response

Below Secondary	☐ 1
Secondary	☐ 2
Post-Secondary (Non-Tertiary)	☐ 3
Diploma and Professional Qualification	☐ 4
University	☐ 5
Post-Graduate Degree	☐ 6
